# Polymorphisms and avascular necrosis in patients with sickle cell disease – A systematic review

**DOI:** 10.1590/1984-0462/2022/40/2021013IN

**Published:** 2022-05-11

**Authors:** Márcio Passos Leandro, Natália Damasceno Almeida, Lara Santana Hocevar, Cloud Kennedy Couto de Sá, Amâncio José de Souza, Marcos Almeida Matos

**Affiliations:** a Hospital Geral Ernesto Simões Filho, Salvador, BA, Brasil.; b Bahia School of Medicine and Public Health, Academic Unit of Brotas, Salvador, BA, Brazil.

**Keywords:** Anemia, sickle cell, Avascular necrosis, Osteonecrosis, Polymorphisms, Doença falciforme, Necrose avascular, Osteonecrose, Polimorfismos

## Abstract

**Objective:**

To systematically establish whether there is an association between polymorphisms and avascular necrosis in patients with sickle cell disease.

**Data source:**

The review, conducted according to PRISMA guidelines and registered with PROSPERO, was based on research of studies in PubMed, SciELO, LILACS, BVS databases and in the gray literature (Google Scholar and Open Gray) published until June 2020. The STROBE initiative was used to analyze the articles’ quality.

**Data synthesis:**

Ten articles were selected from the databases and two were included through manual search, totaling 12 studies. All samples gathered 2,362 patients. According to STROBE, seven studies fully and/or partially covered more than 70% of the essential items and two studies reached less than 60%, with an overall variation of 86.4–54.5%. The results indicate that polymorphisms in the genes of the bone morphogenetic protein 6 (BMP6), Klotho (KL) and Annexin A2 (ANXA2) may be associated with osteonecrosis in the context of sickle cell disease. Six articles addressed the polymorphism in the MTHFR enzyme gene, but only one found a positive association. Polymorphisms associated with the DARC receptor, the ITGA4 gene, CD36 and thrombophilia protein genes were not associated in any of the studies.

**Conclusions:**

The results indicate that the polymorphisms in BMP6, Klotho and ANXA2 genes may be associated with avascular necrosis in patients with sickle cell disease. However, in order to confirm these genetic changes as risk factors, further studies with greater statistical power and methodological rigor are needed.

## Resumo

**Objetivo:**

Estabelecer, de modo sistemático, se existe associação entre polimorfismos e a necrose avascular em pacientes com doença falciforme.

**Fontes de dados:**

A revisão, conduzida segundo as diretrizes *Preferred Reporting Items for Systematic Reviews and Meta-Analyses* (PRISMA) e registrada no *International Prospective Register of Systematic Reviews* (PROSPERO), foi baseada na busca de estudos nas bases de dados PubMed, *Scientific Electronic Library Online* (SciELO), Literatura Latino-Americana e do Caribe em Ciências da Saúde (LILACS), Biblioteca Virtual em Saúde (BVS) e na literatura cinza (Google Scholar e Open Gray) até junho de 2020. A análise da qualidade dos artigos foi baseada nos critérios do Strengthening the Reporting of Observational Studies in Epidemiology (STROBE).

**Síntese dos dados:**

Dez artigos foram selecionados nas bases de dados e dois incluídos por meio da busca manual, totalizando 12 estudos elencados. As amostras resultaram em 2.362 pacientes incluídos. Com base na iniciativa STROBE, sete estudos atenderam total e/ou parcialmente mais de 70% dos itens essenciais e dois atingiram menos que 60% deles, com variação geral de 86,4–54,5%. Os resultados mostram que os polimorfismos nos genes da proteína morfogenética óssea 6 (BMP6), da Klotho (KL) e da Anexina A2 (ANXA2) podem ter associação com osteonecrose no contexto da doença falciforme. Seis artigos estudaram o polimorfismo no gene da enzima MTHFR, mas apenas um obteve associação positiva. Os polimorfismos associados ao receptor DARC, ao gene ITGA4, ao CD36 e aos genes de proteínas trombofílicas não demonstraram associação em nenhum dos estudos.

**Conclusões:**

Os polimorfismos nos genes BMP6, KL e ANXA2 estão possivelmente associados com a necrose avascular em indivíduos com doença falciforme. Entretanto, para a confirmação dessas alterações genéticas como fatores de risco, é necessário que mais estudos com maior poder estatístico e com maior rigor metodológico sejam realizados.

## INTRODUCTION

Sickle cell disease (SCD) is the most common human hereditary hematologic disease, with approximately 300,000 new cases per year worldwide and being of great relevance to the global public health scenario.^1,2^ It is a autosomal recessive hemoglobinopathy caused by a mutation that replaces glutamic acid with valine, producing the abnormal protein hemoglobin S (HbS).^3^ The disease is characterized by deformation of the red cell structure, which acquires a sickle-like shape in low-oxygen tensions.^1^ The sickled structure provides a greater adhesion, facilitating the grouping of red blood cells, compromising blood flow and, consequently, leading to vaso-occlusive crisis (VOC) with serious clinical repercussions.^1,3,4^

Avascular necrosis (AVN) is one of the consequences. This condition is caused by blood flow reduction to the bones^5^, mainly affecting the femoral head and shoulders’, knees’ and ankles’ joints. The pathological changes of this osteonecrosis result in pain, functional limitation of affected limbs, reduced school performance and poorer quality of life.^4,5,6,7,8^ Studies have tried to understand the role of genetic polymorphisms in the development of AVN in patients with SCD.^3^.^9^

Genetic polymorphisms, naturally present in the population, are alterations in the DNA sequence produced through the substitution, deletion or insertion of nitrogenous bases or base sequences. These can culminate in direct modifications in the functioning and expression of proteins or constitute markers indirectly associated with genetic-origin pathological processes.^10,11^ Potential increased risk of AVN has been noted in several polymorphisms described in the literature, involving genes associated with cell growth, nitric oxide metabolism, and coagulation by mechanisms that favor platelet adhesion and aggravate arterial occlusive disease.^9^ Thus, in SCD, these polymorphisms can act as enhancers of endothelial dysfunction, with a loss of the protective effect against oxidative stress, reduction in nitric oxide production, and vaso-occlusive and endothelial changes.^3^

So, it is essential to understand whether studies available in the literature are able to relate the presence of these polymorphisms associated with AVN to SCD. Understanding that bone involvement is an important factor in the worsening of individuals with SCD, identifying specific potential biomarkers can improve the prognostic mechanisms in the course of treatment. The aim of this systematic review was to assess whether there is a relevant association between polymorphisms found in SCD and avascular bone necrosis.

## METHOD

We performed a systematic literature review based on the Preferred Reporting Items for Systematic Reviews and Meta-Analyses (PRISMA) guidelines,^12^ registered in the International Prospective Register of Systematic Reviews (PROSPERO) database under the number CRD42020192074.

Studies addressing polymorphisms and AVN (or osteonecrosis) in SCD were extracted from the databases until June 2020, with no restriction as to language and year of publication, sex or age of participants. Review articles, case reports and other articles in which it was not possible to separate osteonecrosis and other vascular complications in SCD were excluded.

**Chart 1.**
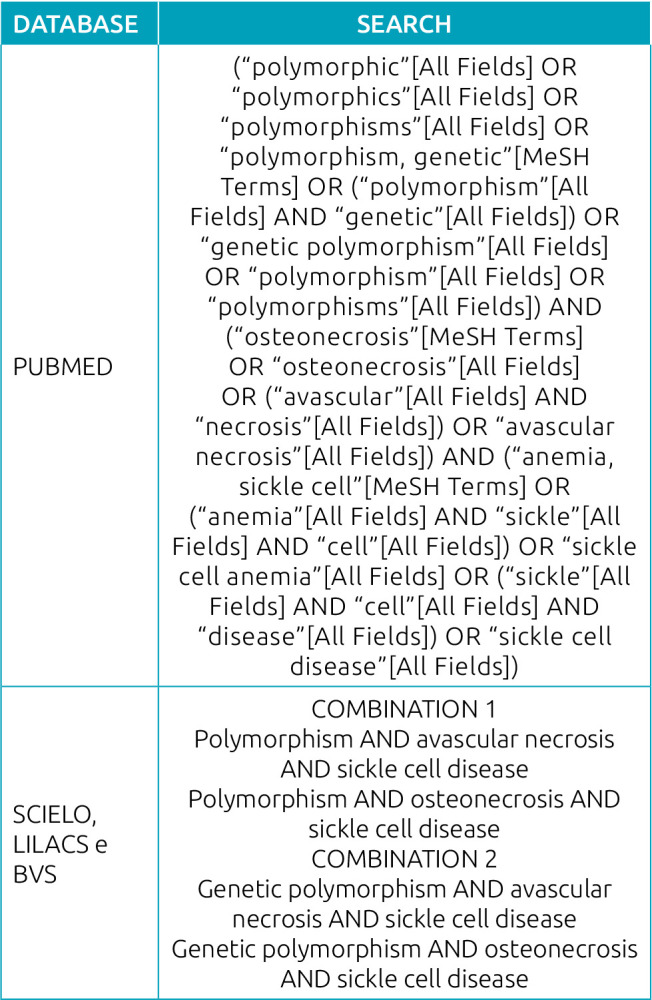
Database search strategies.

A computerized bibliographic search was carried out in the electronic databases PubMed, Scientific Electronic Library Online (SciELO), Latin American and Caribbean Literature in Health Sciences (LILACS), Virtual Health Library (VHL); and in gray literature databases such as Google Scholar, Open Gray. The Medical Subject Headings (MeSH) and Descriptors in Health Sciences (DeCS) were used as a basis, and we used terms corresponding to “polymorphism, genetic”, “osteonecrosis”, “avascular necrosis” and “sickle cell disease”. The terms were combined with the Boolean operators “AND” and “OR” (Chart 1). A manual search was also carried out in the reference list of selected articles. All searches were performed by June 2020. References were managed and duplicate articles were removed.

The works were identified by title and abstract by two independent reviewers (L.S.H and N.D.A.), who followed the inclusion and exclusion criteria. Studies without abstracts whose title suggested meeting the selection criteria were also selected for analysis. All divergences were resolved by consulting a third reviewer (M.P.L.), who finally defined which articles would be fully read. The selected works were read in full by three authors. Then, the studies were included in the systematic review upon agreement of the three reviewers.

Data was extracted by one author (N.D.A.) and verified by another (L.S.H.). Disagreements were resolved through debates. A third author (M.P.L) was involved to make the final decision.

The data collected were: study authors, year of publication, country, study design, mean age, sample size, number of patients, polymorphisms addressed, characteristics of evaluations and classifications, and, finally, conclusions.

Two independent reviewers (L.S.H. and N.D.A.) used their critical appraisal criteria to review all articles included. The checklist proposed by Strengthening the Reporting of Observational Studies in Epidemiology (STROBE)^13^ was applied. The items on the list were classified as: fully met, partially met or not met. The percentage of satisfaction used was the sum of items fully and partially met. Odds Ratio (OR), relative risks (RR), chi-square test, Fisher test, Student’s t test, Mann-Whitney test, Kruskal-Wallis and logistic regression models were used to interpret the results. Clinical, methodological and statistical heterogeneity were explored across studies.

## RESULTS

**Figure 1.**
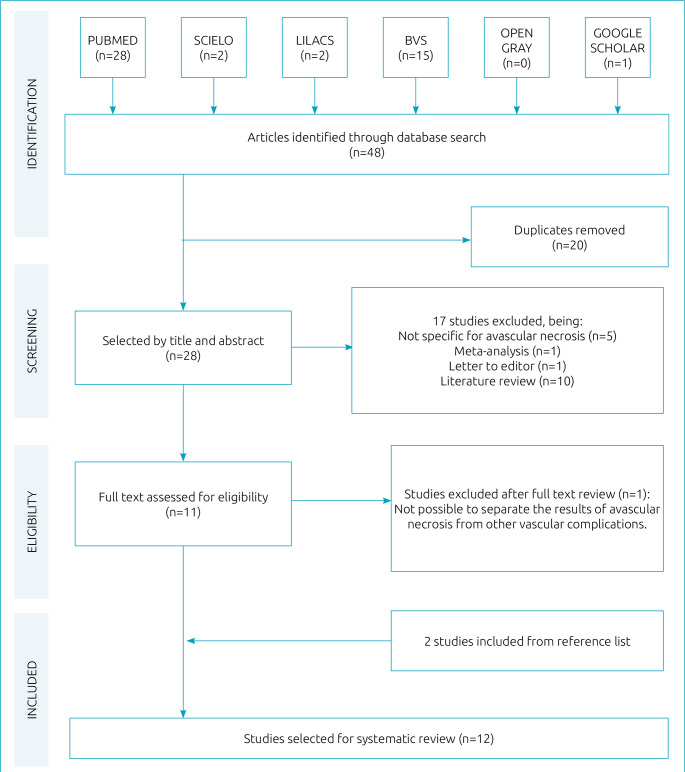
Flowchart of literature search and selection criteria for studies on the association of polymorphisms with osteonecrosis in sickle cell disease.

Upon selection in search platforms and removal of duplicate articles, 28 articles were identified (Figure 1). After analyzing the title and abstract, 17 studies were excluded, so 11 were selected for reading. Of these, one was excluded because it did not differentiate AVN from other vascular complications in data analysis. The manual search included two more articles. At the end, 12 works were selected for full analysis.

BVS: Virtual Health Library.

The selected studies were published between 1998 and 2016, all in English. The 12 articles were classified as cross-sectional. The AVNs identified in people with SCD are mostly classified by a radiological analysis, and polymorphisms and their incidence and associations with AVN in these patients were assessed.

All articles presented a rational justification for their accomplishment, informing the methods used to obtain data, for data analysis, subject evaluations, description of subjects, outcomes and predictors, and discussion of limitations. Four papers claimed to have received support from research intuitions, while the others did not disclose funding sources or reported that the authors themselves funded the research.

Four studies were carried out with the North American population, two with Brazilians, one with Kuwaitis, one with Indians, one with Egyptians, one with Tunisians and one in Guadeloupe (French Antilles) (Table 1). The samples of patients with SCD in whom osteonecrosis was evaluated totaled 2,362 subjects, including pediatric, adult and elderly patients, ranging from 21 to 897 individuals analyzed per article. Of this total, 822 were diagnosed with AVN (mainly of the head of the femur and/or of the head of the humerus).

**Table 1.** Main characteristics of selected articles.

Study	Origin	Study design	Sample	Age	Bones involved	STROBE
Baldwin et al.^15^	USA	Cross-sectional	897 HbSS/442 with AVN/455 without AVN	NR	Hip and/or shoulder	81.8%
Chaouch et al.^18^	Tunisia	Cross-sectional	100 HbSS/81 with AVN/19 without AVN	5–30	Head of femur	72.7%
Kutlar et al.^23^	USA	Cross-sectional	107 HbSS/45 with AVN/62 without AVN	15–54	Head of femur and humerus	54.5%
Pandey et al.^27^	India	Cross-sectional	60 SS /45 Sβ^0^/15 SD/154 controls/14 AVN	4–12	NR	68.2%
Hatzlhofer et al.^14^	Brazil	Cross-sectional	277 SS e Sβ°/177 with VC/100 without VC/ 43 AVN	5–72	Head of femur	86.4%
Nebor et al.^17^	French Antilles	Cross-sectional	212 HbSS/201 with AVN	11–35	Hip or shoulder	81.8%
Moreira Neto et al.^25^	Brazil	Cross-sectional	29 HbSS/24 HbSC/2 with AVN	13–72	Head of femur and humerus	77.3%
Farawela et al.^16^	Egypt	Cross-sectional	59 HbSS /40 HbSβ/8 AVN	2–29	Hip and/or shoulder	81.8%
Kalai et al.^28^	Tunisia	Cross-sectional	66 HbSS/36 HbSβ/11 with AVN	5–12	NR	72.7%
Zimmerman et al.^29^	USA	Cross-sectional	101 HbSS/16 with AVN/85 without AVN	4–62	Head of femur and humerus	68.2%
Zimmerman et al.^30^	USA	Cross-sectional	89 SCD/14 with AVN	5–60	Head of femur and humerus	59.1%
Adekile et al.^31^	Kuwait	Cross-sectional	33HbSS/8 Hbβ0-thal	2–41	Hip	63.3%

USA: United States of America; AVN: avascular bone necrosis; VC: vascular complications; NR: not reported.

Eight studies associated the results with predictors using the chi-square test or Fisher’s exact test, four performed non-parametric tests (Mann-Whitney or Kruskal-Wallis), four used the Student’s t test, one used logistic regression model and four had some risk indicator (OR or RR). One study did not assess risk association or analysis and drew conclusions based on the percentage of results found.

Seven articles met totally or partially more than 70% of the essential items, according to STROBE, ranging from 72 to 86%. Of these, Hatzlhofer et al.,^14^ Baldwin et al.,^15^ Farawela et al.^16^ and Nebor et al.^17^ stood out for the high percentages achieved in STROBE, associated with consistent methodology. The other studies varied between 54.1 and 68.2% in their evaluation (Table 1).

Polymorphisms with associations to AVN were identified in patients with SCD (Table 2). These polymorphisms were found in the genes of bone morphogenetic protein 6 (BMP6) — rs26719, rs267201, rs270393, rs449853 and rs1225934; Klotho (KL) — rs480780, rs211235, rs2149860, rs685417, rs516306, rs565587, rs211239, rs211234, rs499091 and rs576404; and from Annexin A2 (ANXA2) — rs7163836, hCV11770326, rs7170178, rs1033028, hCV26910500 and hCV1571628. Six articles addressed the polymorphism in the gene of the enzyme Methylenetetrahydrofolate reductase (MTHFR), but only one reported a positive association. Polymorphisms associated with the DARC receptor (Duffy antigen/chemokine receptor), the ITGA4 gene (in exons 4, 5 and 6), the CD36 adhesion molecule gene (rs198412) and the thrombophilia protein genes (a-fibrinogen, b -fibrinogen, platelet glycoprotein, factor VII, plasminogen activator inhibitor-1, prothrombin and factor V genes) did not show association with AVN in SCD in any of the studies.

**Table 2.** Main results of selected articles.

Study	Polymorphisms studied	Analysis Measures	Results
Baldwin et al.^15^	BMP6, KL and ANXA2	Multiple logistic regression; OR	For KL, ten polymorphisms were associated with osteonecrosis, for BMP6 five, and, for ANXA2, 6 had the same association (p<0.050)
Chaouch et al.^18^	BMP6	Fisher’s exact test, chi-square test, logistic regression, RR	rs267196 and rs267201 (RR of 1.31) of BMP6 can be considered biomarkers for AVN in SCD.
Kutlar et al.^23^	MTHFR	chi-square test	MTHFR may be associated with AVN in SCD (p=0.006)
Pandey et al.^27^	ANXA2	chi-square test	The polymorphism in the ANXA2 rs7170178 gene was more frequent in patients with osteonecrosis
Hatzlhofer et al.^14^	MTHFR	Fisher’s exact test, chi-square test, OR	No association (p=0,170)
Nebor et al., 2010.	Duffy antigen/chemokine receptor (DARC)	Pearson, Student’s t-test or non-parametric Mann-Whitney test	No association (p=1,000)
Moreira Neto et al.^25^	MTHFR, factor V and prothrombin	Mann-Whitney nonparametric test and Fisher test	No association
Farawela et al.^16^	Duffy antigen/chemokine receptor (DARC)	Fisher’s test, Kruskal-Wallis test and Student’s t test	No association (p=1,000)
Kalai et al.^28^	CD36-rs1984112	Mann-Whitney nonparametric test and Student’s t test	No association (p=1,000).
Zimmerman et al.^29^	Thrombolytic mutations*	Chi-square test, standard error and CART analysis	No association (p>0,050).
Zimmerman et al.^30^	MTHFR and GPilla	Chi-square test and Student’s t test	No association (p>0,050)
Adekile et al.^31^	MTHFR (C677T)	Not informed	The frequency of the MTHFR polymorphism (C677T) was the same with or without AVN (21.4%)

*Thrombolytic mutation of: α-fibrinogen (A312G), β-fibrinogen (G448A), GPIIIa (C1565T), PAI-I (4G), VII Factor (R353Q), MTHFR (C677T) VII Factor repeated sequence (seven repeats), Prothrombin (G20210A) V Factor (G1691A). AVN: avascular bone necrosis; SCD: sickle cell disease; BMP6: bone morphogenetic protein 6; KL: Klotho; ANXA2: annexin A2; MTHFR: methylenetetrahydrofolate reductase; DARC receptor: Duffy antigen/chemokine receptor; GPIIIa: platelet glycoprotein; PAI-1: plasminogen activator inhibitor-1; OR: Odds Ratio; RR: relative risk.

## DISCUSSION

Based on the articles included in this review, the researched data associated AVN in SCD with polymorphisms in the BMP6, KL and ANXA2 genes, which are involved in bone metabolism.^14^ Chaouch et al.^18^ reported that the polymorphisms rs267196 and rs267201 do BMP6 are reliable biomarkers to predict patients at high risk for osteonecrosis.

Regarding the relationship of these genes with bone function, BMP6, which is part of the TGF-β superfamily (transforming growth factor beta), is involved in cell signaling pathways associated with the growth and differentiation of chondrocytes and osteoblasts, being important in bone formation.^16,19^ The KL gene is responsible for functions such as the control of ion channels and endocrine pathways that regulate vitamin D levels, having anti-apoptotic and oxidative stress reducing effects that protect the vascular endothelium and induce the production of nitric oxide (NO).^16,20^ It is believed that the loss of this protection, through alterations in the KL gene and NO reduction, is related to the events that lead to AVN.^20^ ANXA2, which is part of the calcium-regulated phospholipid-binding protein family, is responsible for regulating processes involved with homeostasis, in addition to playing an important role in bone mineralization.^21,22^

Among 12 studies evaluated, six analyzed the MTHFR enzyme. In only one, carried out by Kutlar et al.^23^, a possible specific association of the gene polymorphism with osteonecrosis was found in patients with SCD. The negative findings are similar to what was reported in the meta-analysis conducted by Chai et al.^24^, in which the single nucleotide polymorphism in the MTHFR gene, which promotes the exchange of cytosine for thymine (677C>T), is not related to the development of bone necrosis of the head of the femur, although this was not a study specific for sickle cell patients. However, Moreira Neto et al.^25^ and Hatzlhofer et al.^14^ stated that this polymorphism was associated with a set of vascular complications (acute chest syndrome, infarction, priapism, ulcers in the lower limbs and osteonecrosis) commonly present in SCD. This relationship was also reported in the meta-analysis carried out by Lakkakula,^26^ whose conclusion was a positive association between polymorphism in the MTHFR gene and an increased risk of vascular complications in individuals with SCD. However, it was not possible to analyze each of these comorbidities individually.

An exhaustive search was made to build this review in an attempt to also include the “grey literature”. There is a low probability of publication bias in view of this strategy and the analysis of included studies, considering methodological and statistical criteria. One must also consider the language barrier in publications, in view of the high incidence of SCD in Africans and Asians and the tendency of cases published in respective languages. Finally, an important limitation in this review is the fact that most do not report how the sample size was determined, which increases the probability of false negatives due to the possible lack of statistical power to demonstrate associations.

In conclusion, there are genetic polymorphisms that are possibly associated with avascular bone necrosis in individuals with SCD. Mutations in genes BMP6, ANXA2 and KL are the most evident according to the results obtained.
